# The Heterologous Expression of the Chrysanthemum R2R3-MYB Transcription Factor *CmMYB1* Alters Lignin Composition and Represses Flavonoid Synthesis in *Arabidopsis thaliana*

**DOI:** 10.1371/journal.pone.0065680

**Published:** 2013-06-19

**Authors:** Lu Zhu, Hong Shan, Sumei Chen, Jiafu Jiang, Chunsun Gu, Guoqin Zhou, Yu Chen, Aiping Song, Fadi Chen

**Affiliations:** 1 College of Horticulture, Nanjing Agricultural University, Nanjing, China; 2 Institute of Fishery Science of Nanjing, Nanjing, China; Lawrence Berkeley National Laboratory, United States of America

## Abstract

Plant R2R3-MYB transcription factor genes are widely distributed in higher plants and play important roles in the regulation of many secondary metabolites at the transcriptional level. In this study, a chrysanthemum subgroup 4 R2R3-MYB transcription factor gene, designated *CmMYB1*, was isolated through screening chrysanthemum EST (expressed sequence tag) libraries and using rapid application of cDNA ends (RACE) methods and functionally characterized. *CmMYB1* is expressed in the root, stem, leaf and flowers, but most strongly in the stem and most weakly in the root. Its heterologous expression in *Arabidopsis thaliana* reduced the lignin content and altered the lignin composition. The heterologous expression also repressed the flavonoids content in *A. thaliana*. Together, these results suggested that *CmMYB1* is a negative regulator of genes involved in the lignin pathway and flavonoid pathway, it may be a promising gene for controlling lignin and flavonoids profiles in plants.

## Introduction

Transcription factors play critical roles in regulating plant development and response to environmental stress. The structure of the DNA binding sequences of transcription factors has allowed the recognition of several distinct gene families [1]. In plants, MYB family is one of the most abundant transcription factor classes [2]. Among approximately 1500 transcription factor genes identified in the *Arabidopsis thaliana* genome, 130 belong to the MYB members [3], [4]. The MYB DNA binding domain consists of one to three imperfect 50–53 residue repeats [5], and the number of these repeats present has been used to categorize the family into the sub-classes 3R-MYB, R2R3-MYB and a MYB-related group; of these, R2R3-MYB is the most commonly occurring type [6], [7]. Based on variation within certain conserved C-terminal motifs, the R2R3-MYBs have been classified into 22 sub-groups [8], with members within each sub-group predicted to share similar or identical functions [9].

The complex polyphenol molecule lignin is deposited in the secondary cell walls of all vascular plants and is synthesized through the lignin biosynthesis pathway, a major branch of the phenylpropanoid pathway [10]. Its accumulation improves the efficiency of water transport, increases the stiffness of mechanical tissues, and forms a physical barrier against pathogens and wounding [10]–[12]. The lignin synthesis is heavily regulated by R2R3-MYB transcription factors [13], [14]. Several R2R3-MYB transcription factors belonging to different subgroups such as *Arabidopsis AtMYBPAP1* [15], *Pinus taeda PtMYB1* and *PtMYB4* [16], [17], *poplar PttMYB21a* [18], *Eucalyptus gunnii EgMYB2* [19] and *Vitis vinifera VvMYB5a* [20] have already been shown to either positively or negatively control the lignin biosynthesis through their *in vitro* interaction with ACI, ACII, and ACIII cis-elements. So far, all the repressors of lignin synthesis appear to be confined to subgroup 4. For example, the *Antirrhinum majus AmMYB308* and *AmMYB330* were the first R2R3-MYB factors associated with the down-regulation of lignification. When either of the MYB proteins was heterologously expressed in tobacco, the vascular tissue lignin content was markedly reduced, and transcript abundance of the genes encoding 4-coumarate:CoA ligase (*4CL1*), cinnamate-4-hydroxylase (*C4H*) and cinnamyl alcohol dehydrogenase (*CAD*) was reduced [21]. Similarly, *C4H* expression was enhanced in the *A. thaliana* knock-out mutant *Atmyb4* while that of *CCoAOMT* (*caffeoyl-CoAO-methyltransferase*) was reduced [22]; meanwhile in the *Atmyb32* mutant, the gene encoding caffeic acid O-methyl-transferase (*COMT*) was up-regulated [23]. The two *Zea mays* subgroup 4 R2R3-MYB factors *ZmMYB31* and *ZmMYB42* also act as repressors of lignin synthesis [24], [25], and the heterologous expression of *ZmMYB42* in *A. thaliana* appears to reduce lignin content, alter lignin composition and repress flavonoids biosynthesis [26]. Similarly, the over-expression of *ZmMYB31* significantly reduces lignin content with alter polymer composition, and enhances *CHI, F3H, F3*′*H* and *DFR* gene expression in *A. thaliana* [25]. Finally, *EgMYB1* expression reduces lignin synthesis in both transgenic *A. thaliana* and *P. trichocarpa* [27], [28].

Chrysanthemum (*Chrysanthemum morifolium*) is a commercially important ornamental plant worldwide. Here, we describe the isolation of the subgroup 4 R2R3-MYB transcription factor *CmMYB1.* Its participation in the regulation of lignin synthesis and flavonoid synthesis was demonstrated by its heterologous expression in *A. thaliana*. A series of gene expression experiments demonstrated that several genes in the lignin synthesis pathway and flavonoid biosynthesis were down-regulated by the presence of the *CmMYB1* transgene, leading to a reduction in the lignin content, lignin compositions as well as a decrease in the flavonoids content in the transgenic plants.

## Materials and Methods

### Plant Materials and Plant Growing Conditions

The chrysanthemum variety ‘Zhongshanzigui’ was obtained from the Chrysanthemum Germplasm Resource Preserving Centre, Nanjing Agricultural University, China. Plants were grown in a 1∶1 mixture of garden soil and vermiculite without any additional fertilizer, and were maintained in a greenhouse under standard growing conditions. Young plants were watered daily, and fertilized weekly with half strength Hoagland’s nutrient solution. The expression profile of *CmMYB1* was determined in the root, stem, young leaf and ray floret of each of three chrysanthemum plants, using RNA extracted from snap-frozen fresh plant material. *A. thaliana* ecotype Col-0 plants were grown in a 1∶1∶1 mixture of perlite:vermiculite:soilrite in a growth chamber set to deliver a 16 h photoperiod at 23°C during the lit period (80–100 µmol m^−2^ s^−1^ illumination) and 18°C during the dark period. The plants were watered every 4 days. To determine the expression level of various lignin synthetic genes, RNA was extracted from snap-frozen intact plants of three week old whole plants Col-0 and transgenic *A. thaliana* plants.

### Full-length cDNA Isolation and Sequencing

Total RNA was isolated from chrysanthemum leaves using the RNAiso reagent (TaKaRa, Japan), following the manufacturer’s instructions. The cDNA first strand was synthesized from 1 µg total RNA using SuperScript III reverse transcriptase (Invitrogen, USA), according to the manufacturer’s instructions. A gene-specific primer pair (F/R) was designed to amplify a fragment of *CmMYB1* based on the sequence of a chrysanthemum EST (DK942906) [29], and RACE PCR was then used to obtain the full length cDNA. For the 3′ RACE reaction, the first strand was synthesized using an oligo (dT) primer incorporating the sequence of the adaptor primer. Then a nested PCR was employed, using the gene-specific primer pair GSP3′-1/−2 and the adaptor primer (dT-AP). For the 5′ RACE reaction, the nested PCR was based on the 5′ RACE adaptor primer (Abridged Anchor Primer, AAP), the Abridged Universal Amplification Primer (AUAP) provided with the 5′ RACE System kit v2.0 (Invitrogen) and the gene-specific primers (GSP5′-1, GSP5′-2 and GSP5′-3). PCR products were purified using a Biospin Gel Extraction kit (Bio Flux) and cloned into the pMD19-T easy plasmid (TaKaRa) for DNA sequencing. Finally, a pair of gene-specific primers (Full-F/Full-R), designed from the putative 5′ and 3′ UTR sequences, was used to amplify the complete *CmMYB1* open reading frame. The sequences of all the above primers are given in table S1 in file S1. Chrysanthemum genomic DNA was isolated from young leaves using a CTAB method [30], and the genomic sequence of *CmMYB1* was amplified using the primer pair Full-F/Full-R. The resulting product was purified using a Biospin Gel Extraction kit and cloned into pMD19-T easy for sequencing.

### Sequence Alignment and Phylogenetic Analysis of *CmMYB1*

The *CmMYB1* peptide sequence was aligned with that of its presumed homologues using ClustalW [31]. A neighbour-joining based phylogenetic tree was constructed using MEGA5 [32].

### Quantitative Real-time PCR (qRT-PCR)

*CmMYB1* expression profiles were inferred from qRT-PCR outputs. Total RNA was extracted using the RNAiso reagent (TaKaRa, Japan), treated with DNaseI to remove any contaminating genomic DNA and converted into cDNA using SuperScript III reverse transcriptase (Invitrogen, USA). The qRT-PCR used SYBR® Green I (TOYOBO, Japan). The primer pair MYB1-RT-F/−R (table S1 in file S1) was applied to amplify a 178 bp fragment in the 3′ region of the gene, avoiding the more well-conserved segments of the gene. A portion of the chrysanthemum *GAPDH* sequence (DK941612), amplified with primers CmGAPDH-F/−R, was used as a reference. Each 25 µl qRT-PCR contained 10 µl SYBR Green PCR master mix, 0.2 µM of each primer and 10 ng cDNA, and the amplification regime consisted of an initial denaturation of 95°C/60 s, followed by 40 cycles of 95°C/15 s, 60°C/15 s, 72°C/45 s. The resulting data are given as means ± SE of three biological replicates. Relative expression levels were calculated based on the 2^−△△CT^ method [33], [34]. The expression levels of genes involved in the synthesis of lignin, cellulose and xylan, and flavonoid were also derived by qRT-PCR primers of *PAL1*(AT1G01860), *HCT*(AT5G48930), *C3H*(AT1G01350), *CCR1*(AT1G15950), *F5H* (AT4G36220), *CesA4*(AT5G44030), *CesA7*(AT5G17420), *CesA8*(AT4G18780), *IRX8*(AT5G54690) and *IRX9*(AT2G37090) genes primers were from Bhargava [35], those of *CHS*(AT5G13930), *CHI* (AT2G43570), *F3H*(AT3G51240.2), *F3*′*H*(AT3G51240.1) and *DFR*(AT5G42800) primers were from Zhu [36], all the primers were detailed in table S2 in file S1. RNA was harvested from Col-0 and *CmMYB1* transgenic *A. thaliana* leaves. A portion of the *A. thaliana AtUBQ* gene (NM_116771.5), amplified by primer pair AtUBQ-F/−R, was used as the reference.

### Vector Construction and *A. thaliana* Transformation

The *CmMYB1* coding sequence was amplified using a forward primer (MYB1-1301-F) incorporating a *Bam*H I restriction site and a reverse primer (MYB1-1301-R) with a *Sac* I site (table S1 in file S1). *Bam*H I*-Sac* I digested amplicons were inserted into pCAMBIA1301 to generate a *35S::CmMYB1* construct, which was introduced into Col-0 via an *Agrobacterium tumefaciens EHA105*-mediated ﬂoral dip method [37]. Transformed progeny were selected by germination on a standard medium containing 50 µg mL^−1^ hygromycin, and confirmed by subsequent RT-PCR analysis.

### Lignin Analysis

Stem cross-sections of ten *35S::CmMYB1* transgenic *A. thaliana* plants and ten wild type plants were prepared using a scalpel blade, respectively. Sections of thickness 70 µm were mounted on a microscope slide and kept moist by the addition of distilled water. After removal of all excess water, a few drops of 6 mol^.^L^−1^ HCl were placed on the sections and left for 3 min. Thereafter, a few drops of 5% (w/v) phloroglucinol-HCl were added and the sample covered with a cover slip for 10 min [38]. Phloroglucinol-HCl reacts with the hydroxycinnamaldehyde and benzaldehyde groups of lignin, and the intensity of the red stain generated by this reaction roughly reflects total lignin content [39]. The samples were inspected by light microscopy.

Total lignin content was determined by the spectrophotometric acetyl bromide lignin method with modifications [40]. The samples were determined for three biological replicates, each with 60 mg of drying stem. The cell walls of samples were isolated and then extracted in 100 µl acetyl bromide (25% v/v acetyl bromide in glacial acetic acid) at 50°C for 2 h, and cooled on ice to room temperature. Subsequently, add 400 µl 2 mol^.^L^−1^ NaOH and 70 µl of freshly prepared 0.5 mol^.^L^−1^ hydroxylamine hydrochloride to the cooled samples, vortex volumetric flasks. Fill up volumetric flask exactly to the 2 ml with glacial acetic acid, cap and invert several times to mix and the content was measured through spectrophotofluorometer at 280 nm. The lignin monomer composition from *A. thaliana* mature stems was measured with the CuO oxidation method by HPLC [41].The HPLC separation procedures followed protocol of Sonbol FM et al [26].

### Cellulose Content Analysis

Method for measuring the content of cellulose is essentially described by Updegraf [42]. Briefly, 60 mg dried cell wall was added to 1 ml Updegraff reagent (Acetic acid: nitric acid: water, 8∶1∶2 v/v) and heated at 100°C for 30 min. The pellets were washed with water and acetone and added to 72% sulfuric acid to hydrolyze completely. The glucose content was read at 625 nm.

### Flavonoids Analysis by HPLC

HPLC analysis was performed using method of Burbulis [43] with minor revision. For flavonoids analysis, 100 mg fresh mature stems were harvested, and then extracted in 500 µl methanol with thoroughly vortexing, and centrifuged for 10 min at 10,000 rpm. The supernatant was hydrolyzed with 2 mol^.^L^−1^ HCl at 70°C for 40 min. After being cooled in room temperature, the hydrolysis were terminated by adding 500 µl methanol, the mixture was dried under N_2_ stream and re-dissolved with 500 µl methanol for the HPLC analysis. The separation and quantification of flavonoids were determined in following conditions. Injected volume: 20 µl. Column: ZORBAX Eclipse Plus C18 of 5 µm, 4.6×250 mm. Eluents: (a) 0.05% acetic acid, (b) methanol. Flux: 1 ml/min. Temperature: 25°C. The gradient started with 10% methanol, increasing to 50% in 10 min, 90% in 20 min, 100% in 5 min. Flavonoids (kaempferol, quercetin and isorhamnetin) were detected at 254 nm.

## Results

### Isolation of *CmMYB1*, a new Subgroup 4 R2R3-MYB Factor from *chrysanthemum*

**Figure 1:**
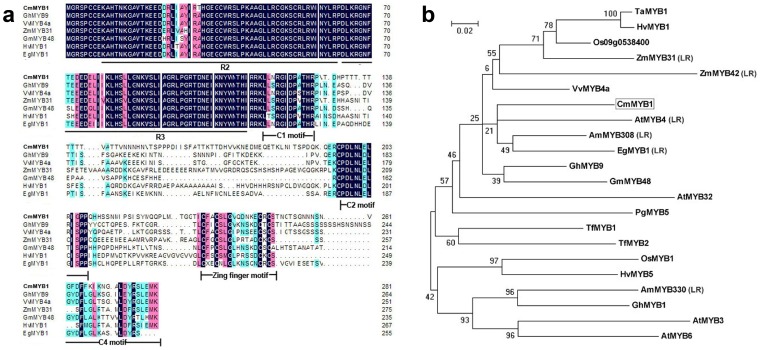
The deduced peptide sequence of *CmMYB1* (marked in bold) and related MYBs. a. Peptide alignment. R2 and R3 MYB DNA binding domains are shown underlined. C1 motif: LLsrGIDPX[T/S]HRX[I/L]. C2 motif: pdLNL[D/E]LXi[G/S]. C4 motif: GYDFLG[L/M]X_4–7_L_X_[Y/F][R/S]XLEMK. Zing finger motif: CX_1–2_CX_7–12_CX_1–2_C; b. The phylogeny of *CmMYB1* and related MYBs. Bootstrap values of each branch of the derived tree are given, and the scale bar represents 0.02 substitutions per site. The genes encoding the amino acid sequences and their GenBank accession numbers are: *VvMYB4a* (XP_002278222), *GhMYB9* (AAK19619), *ZmMYB31* (NP_001105949), *AtMYB4* (AAS10085), *AmMYB308* (P81393), *GmMYB48* (ABH02823), *TaMYB1* (AAT37167), *Os09g0538400* (NP_001063796), *OsMYB1* (BAA23337), *AtMYB32* (NP_195225), *PgMYB5* (ABQ51221), *HvMYB1* (P20026), *HvMYB5* (CAA50221), *TfMYB1* (AAS19475), *ZmMYB42* (NP_001106009), *AmMYB330* (P81395), *AtMYB3* (NP_564176), *AtMYB6* (NP_192684), *EgMYB1* (CAE09058), *GhMYB1* (AAN28270), *TfMYB2* (AAS19476). *CmMYB1* is in bold. LR: R2R3-MYB transcription factors characterized as repressors of lignin synthesis.

The full length cDNA *CmMYB1* sequence (JF795917) was isolated by RT-PCR and RACE based on an EST created by Chen [29]. It consisted of 1, 237 nucleotides, of which 846 bp represented an open reading frame encoding 281 residues. The predicted gene product is a protein of molecular mass 31.7 kDa and a pI of 8.77, containing an R2R3 MYB domain with two DNA binding sites, one localized between residues 9 and 61, and the other between residues 62 and 116. Four conserved regions lie at its C-terminus, namely LLsrGIDPX[T/S]HRX[I/L], pdLNL[D/E]LXi[G/S], GYDFLG[L/M]X_4–7_L_X_[Y/F][R/S]XLEMK, and CX_1–2_CX_7–12_CX_1–2_C; these are, respectively, putative C1, C2, C4 and zing finger motifs, unique to subgroup 4 R2R3-MYB genes [8]. C2, which contains the core EAR-motif, plays a major role in gene repression [44], [45]. The alignment of the *CmMYB1* sequence with those of homologous R2R3-MYB proteins showed levels of similarity ranging between 55% and 62% (Fig. 1a), and suggested a close phylogenetic relationship with the well known subgroup 4 lignin repressors *AmMYB308*, *AmMYB330*, *EgMYB1* and *ZmMYB31* [21], [25], [27] (Fig. 1b). Comparison of the *CmMYB1* genomic DNA and cDNA sequences identified the presence of a single 307 bp intron, the position of the intron (264 bp downstream of the ATG start codon) is well conserved among R2R3-MYB subgroup 4 genes (data not shown).

### *CmMYB1* Expression Profiles in *chrysanthemum*

**Figure 2:**
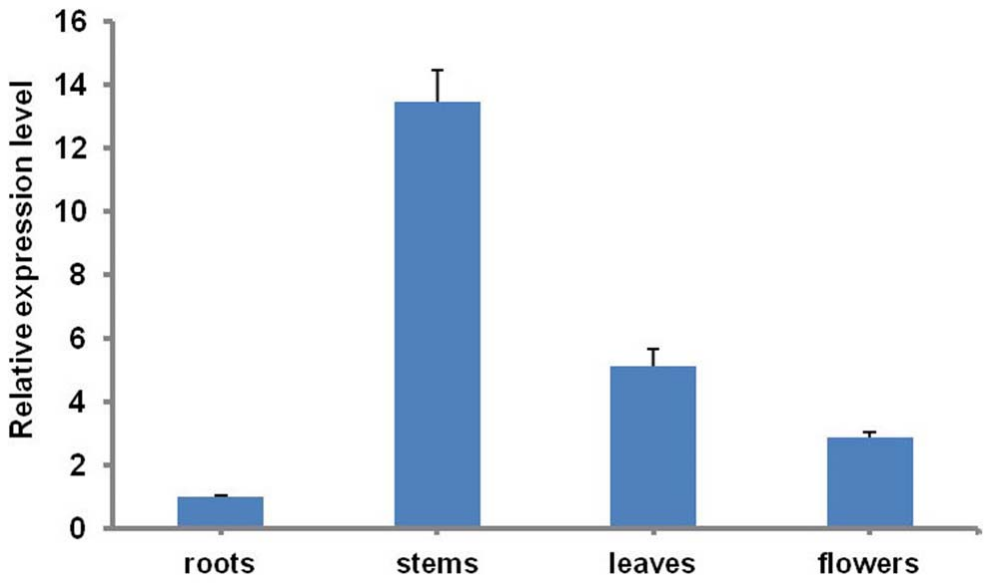
The expression of *CmMYB1* in various chrysanthemum tissues. Values shown are means ± SE, as calculated from three biological replicates.

To gain the expression pattern of *CmMYB1*, qRT-PCR was carried out to analyze samples from various organs of the chrysanthemum plant. The results showed that *CmMYB1* was ubiquitously expressed with the strongest expression level in stems. *CmMYB1* was also expressed at a lower level in leaves, as well as in flowers, and the weakest level in roots (Fig. 2).

### The Effect of Heterologous Expression of *CmMYB1* on *A. thaliana*

**Figure 3:**
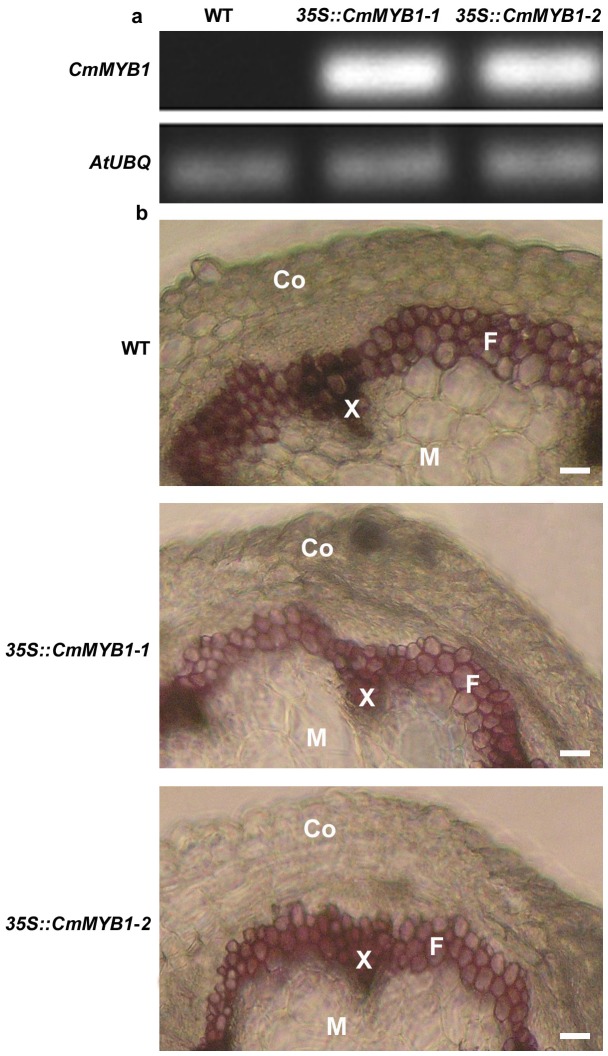
Lignin synthesis in transgenic and wild type *A. thaliana* plants. a. RT-PCR demonstrates the heterologous expression of *CmMYB1* in the transgenic lines. The *AtUBQ* sequence was used as an internal control; b. The histochemical detection of lignin in wild type Col-0 (WT) and transgenic *A. thaliana* plants expressing *CmMYB1* (35S::*CmMYB1*-*1* and 35S::*CmMYB1*-*2*). Lignified tissues are stained red with phloroglucinol-HCl. The image shown is representative of ten observations. Co: cortex; F: interfascicular fibres; X: xylem; M: medular parenchyma.

*CmMYB1* was introduced under the control of the cauliflower mosaic virus 35S promoter into binary vector pCAMBIA1301 containing the hygromycin B and gusA as selectable markers. Transformation was performed using floral dip method [37]. Two independent transgenic *A. thaliana* lines (*35S::CmMYB1-1*, *35S::CmMYB1-2*) which showed higher levels of transgene expression were selected from several transgenic lines for further experiments (Fig. 3a). There was no observable phenotypic difference between wild type and transgenic plants grown under long days (16 h light and 8 h dark). The phloroglucinol staining generated red coloration uniformly distributed throughout the lignified tissues of both the wild type and the transgenic plants (Fig. 3b), but the staining intensity was noticeably greater in the wild type stems than in those of either of the two transgenic lines.

### *CmMYB1* Affects Lignin Biosynthesis

**Figure 4:**
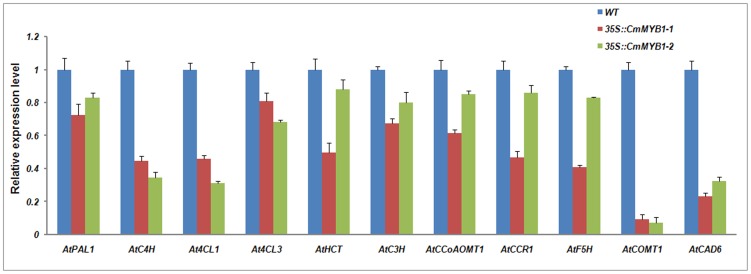
Expression profiles of genes involved in lignin synthesis in wild type and transgenic *A. thaliana* plants heterologously expressing *CmMYB1*. Values shown are means ± SE, as calculated from three biological replicates.

The expression level of a set of key lignin synthesis genes (*AtPAL1, AtC4H*, *At4CL1*, *AtHCT, AtC3H, AtCCoAOMT1*, *AtCCR1, AtF5H, AtCOMT1* and *AtCAD6*) was monitored in the transgenic lines using qRT-PCR, respectively. This experiment showed that the expression of *AtCOMT1 and AtCAD6* had been decreased to 10% of the wild type level and that of *AtC4H*, *At4CL1, AtHCT, AtCCR1,* and *AtF5H* to between 25% and 50%. However, the level of *AtPAL1, At4CL3*, *AtC3H* and *AtCCoAOMT1* expression appeared to have been less affected (Fig. 4).

**Figure 5:**
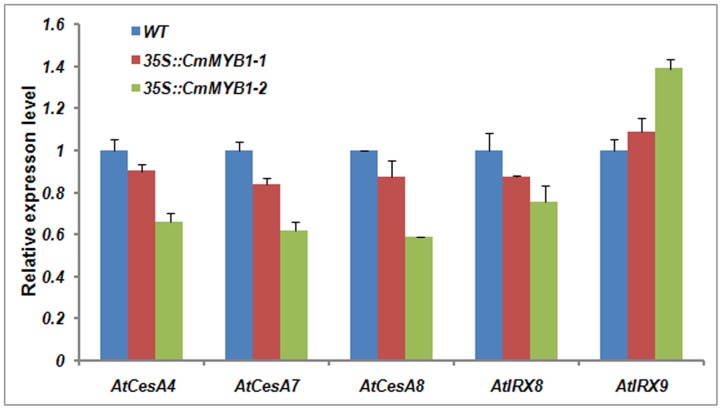
Heterologously expressing *CmMYB1* does affect the expression of secondary wall–associated cellulose synthase genes (*CesA4, CesA7, and CesA8*) and xylan biosynthetic gene *IRX9*, but does not induce another xylan biosynthetic gene *IRX8*. Values shown are means ± SE, as calculated from three biological replicates.

The expression level of genes involved in the biosynthesis of cellulose was also affected by heterologous expression of *CmMYB1*. qRT-PCR analysis showed that the expression of three secondary wall–associated cellulose synthase genes (*CesA4, CesA7, and CesA8*) [46], [47] had been decreased. But that of xylan biosynthetic genes *IRX8* [48] had been less down-regulated. The level of another gene of xylan biosynthetic *IRX9* expression, however, was increased (Fig. 5).

To better determine the changes of lignin in two independent transgenic *A. thaliana* (*35S::CmMYB1-1*, *35S::CmMYB1-2*), we assayed the content of acetyl bromide lignin in stems. The analysis revealed that the acetyl bromide lignin content decreased in stems of both the two transgenic plants. The lignin content of transgenic plant *CmMYB1-1* and *CmMYB1-2* had been decreased by 10% and 20% of the wild type plant, respectively (Table 1).We also analysed the lignin monomer composition. An increase in H subunits was observed in two independent transgenic *A. thaliana* plants. The content of G subunits increased significantly by 40% of the wild type while the content of S subunits decreased significantly by 40% of the wild type (Table 1).

**Table 1:** Lignin content in the stems of wild type and heterologous expression of *CmMYB1* on *A. thaliana.*

	Wild type	*35S::CmMYB1-1*	*35S::CmMYB1-2*
**Lignin content(mg/g)**	217.7±0.42	193.6±0.91*	171.0±0.27**
**H** a **(mg/g)**	0.049±0.01	0.48±0.34	0.24±0.01
**G** b **(mg/g)**	1.45±0.82	1.88±0.76*	2.06±0.67**
**S** c **(mg/g)**	2.27±0.05	1.27±0.16**	1.85±0.79*
**S/G**	1.57	0.67	0.90

a refers to p-hydroxybenzaldehyde;

b refers to the sum of vanillic acid and vanillin;

c refers to the sum of syringic acid and syringaldehyde. Values shown are means ± SE, as calculated from three biological replicates. * and **, significant differences (respectively, *P*<0.05 and *P*<0.01).

Cellulose is another important secondary wall component. We also performed analysis of cellulose content, however, no significant reduction in cellulose was observed (Table 2).

**Table 2:** Cellulose content in the stems of wild type and heterologous expression of *CmMYB1* on *A. thaliana.*

Plant	Cellulose content(mg/g dry cell wall)
**Wild type**	285.77±11.92
***35S::CmMYB1-1***	264.36±8.43
***35S::CmMYB1-2***	230.58±14.39

Cellulose content from stems of wild type and heterologous expression of *CmMYB1* (*35S::CmMYB1-1*, *35S::CmMYB1-2*) on *A. thaliana*. Values shown are means ± SE, as calculated from three biological replicates.

### *CmMYB1* Suppresses Flavonoid Biosynthesis

**Figure 6:**
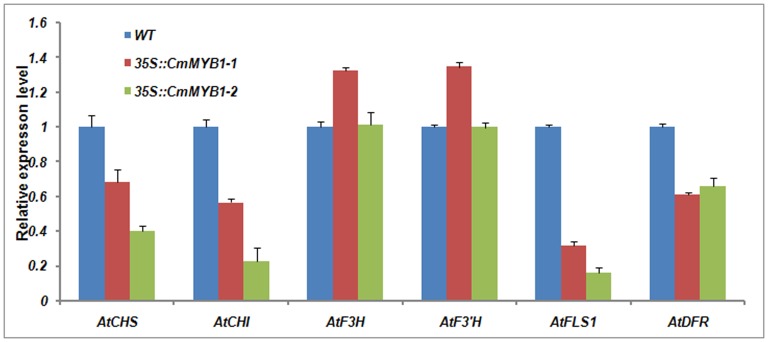
Expression profiles of genes involved in flavonoids synthesis in wild type and transgenic *A. thaliana* plants heterologously expressing *CmMYB1*. Values shown are means ± SE, as calculated from three biological replicates.

We performed qRT-PCR assays to determine the effect of *CmMYB1* on flavonoids biosynthesis. The analysis indicated that the expression of *CHS*, *CHI*, *FLS1* and *DFR* was suppressed, while *F3H* and *F3*′*H* increased slightly in transgenic plants compared to that of the wild type plants (Fig. 6).

Flavonoids content in wild type and two independent transgenic plants of *A. thaliana* (*35S::CmMYB1-1*, *35S::CmMYB1-2*) were determined via HPLC analysis. The contents of quercetin, kaempferol and isorhamnetin of transgenic plant *CmMYB1-1* and *CmMYB1-2* decreased by 60% and 50% of the wild type plant, respectively, which inferred that *CmMYB1* might suppress the flavonoid synthesis (Table 3).

**Table 3:** Flavonoids content in the stems of wild type and heterologous expression of *CmMYB1* on *A. thaliana.*

Plant	Flavonols content (mg/g fresh wt)		
	Quercetin	Kaempferol	Isorhamnetin
**Wild type**	1.094±0.082	0.732±0.0	0.212±0.030
***35S::CmMYB1-1*** ***35S::CmMYB1-2***	0.391±0.012**0.558±0.031**	0.295±0.038**0.387±0.010**	0.101±0.004**0.099±0.006**

Flavonoids content was performed by HPLC from stems of wild type and heterologous expression of *CmMYB1* (*35S::CmMYB1-1*, *35S::CmMYB1-2*). Kaempferol, quercetin and isorhamnetin were detected at 254 nm. Values shown are means ± SE, as calculated from three biological replicates.

** Significant differences (*P*<0.01).

## Discussion

*MYB* genes are a particularly abundant class of plant transcription factors, and the R2R3-MYB subfamily is prominent in higher plant species. Here we have described the isolation of the chrysanthemum R2R3-MYB transcription factor *CmMYB1*. Its sequence alignment and phylogeny strongly indicated that it belongs to R2R3-MYB subgroup 4, which includes a number of members involved in the repression of lignin synthesis [21], [22], [24], [28]. It includes the C2 motif known to confer transcriptional repressor activity [45], which supports the idea that *CmMYB1* functions as a negative regulator in chrysanthemum. *CmMYB1* was ubiquitously expressed in the plant, although most strongly in the stem, which implies that it may play a role in the lignifying tissue of chrysanthemum.

A number of studies have shown that the over-expression of R2R3-MYB factors known to act as repressors of lignin synthesis produce alternations of the leaf morphology, with the appearance of white lesions on mature leaves and a reduction in growth rate when heterologous-expressed in tobacco and over-expressed in Arabidopsis [21], [22]. However, in the present study the heterologous expression of *CmMYB1* in *A. thaliana* did not produce any observable phenotype, which is different from previous researches. To determine whether *CmMYB1* gene was related to the synthesis of lignin, we employed a direct histochemical staining method to detect the lignin content in stem, where the color intensity generated in the reaction with phloroglucinol was enhanced with the increase of lignin content. By this method, the strong reduction of lignin content was observed in inflorescence stems of *CmMYB1* transgenic *A. thaliana* plants. We also employed the acetyl bromide lignin method to determine the lignin content in stems. By this quantitative method, we could confirm the lignin content of transgenic *A. thaliana* plants was decreased (Table 1). In addition, *CmMYB1* overexpression in *A. thaliana* plants altered the lignin composition, showing higher content of H and G subunits while lower content of S subunits characterized by decreased S/G ratio of the lignin. Lignin synthesis was known to interact with cellulose and other secondary cell wall synthesis [26], however, cellulose synthesis was less affected in *CmMYB1* transgenic plants (Table 2).

*PAL* is the first enzyme of the phenylpropanoid pathway. Over-expression of *PtMYB4* in tobacco plants could reduce the expression level of *PAL* [17]. *4CL* genes play key roles in the metabolic pathway of lignin synthesis, catalyzing the reaction for lignin synthesis. Most vascular plants (including *A. thaliana*) possess three distinct *4CL* forms [49]. In *A. thaliana*, *At4CL1* and *At4CL2* are largely responsible for controlling the branching of the growing lignin molecule, while *At4CL3* controls flavonoid branching. A common feature between tobacco heterologous expressing *AmMYB308* or *AmMYB330* and *A. thaliana* over-expressing *AtMYB4* is the down-regulation of *4CL1* expression, as also displayed in the transgenic *A. thaliana* lines expressing *CmMYB1* (Fig. 4); this commonality indicates that *CmMYB1* is involved in lignin synthesis. *CAD* is another key gene in lignin synthesis of *A. thaliana* stems [50], involved in the final step of the reduction reaction of lignin monomer. The lignin content in the stem of double mutant plant (*cad-c, cad-d*) was reduced by 40%, compared to that of normal species, so the stems of *A. thaliana* double mutant were soft and easy to lodge. Therefore, heterologous expression of *CmMYB1 on A. thaliana* reduced the expression of key enzymes *AtCAD* and affected lignin synthesis (Fig. 4). In addition to *4CL1* and *CAD*, subgroup 4 R2R3-MYB factors have also been reported to regulate other key genes in the lignin pathways. *AmMYB308* and *AmMYB330* both negatively regulate the expression of *C4H* and *CAD* when heterologously over-expressed in tobacco [21], and *AtMYB4* is proved to be function as a repressor, particularly of *C4H* [22]. On the other hand, *ZmMYB31* marginally enhances *CAD* expression and decreases *COMT* expression, but does not affect the expression of either *C4H* or *CCoAOMT*, while *ZmMYB42* down-regulates the expression of *C4H*, *CAD* and *COMT* but does not affect the expression of *CCoAOMT* [24]. Here, similar to the behavior of both *AmMYB308* and *ZmMYB42*, the heterologous expression of *CmMYB1* suppressed the expression of *C4H*, *COMT* and *CAD*, while that of *CCoAOMT* was unaffected (Fig. 4).It has been reported that the down-regulated expression of *C3H, 4CL1, F5H* or *COMT* could alter the final lignin composition [51], [52]. *CmMYB1* involved in the expression of lignin biosynthetic genes suggests the process that *A. thaliana* biosynthetic pathways lead to the biosynthesis of cellulose, xylan, and lignin may be cogitated by pathway-specific transcription factors. *CesA* genes play an important part in cellulose biosynthesis [46], [47] and *IRX8* and *IRX9* genes involved in xylan biosynthesis [48]. These results suggest that *CmMYB1* is a transcription factor influencing cellulose biosynthesis, but appeared to have been largely unaffected in xylan biosynthesis (Fig. 5).

*CmMYB1* seems to play an important role in regulating flavonoid pathway which is another branch of phenylpropanoid pathway. *VvMYBF1*, a R2R3-MYB transcription factor, is a functional regulator of flavonol synthesis [53]. *ZmMYB42* repress the flavonol biosynthesis [26]. *AtMYB4* of R2R3-MYB factor also affected the expression level of *4CL3* and *CHS* [22]. Over-expression of *CmMYB1* in *A. thaliana* reduced the expression level of *4CL3*, *CHS*, *CHI*, *FLS* and *DFR*. The contents of quercetin, kaempferol and isorhamnetin contents decreased significantly in consistence with the suppression of genes expression in two independent transgenic *A. thaliana*. Overall, the data indicate that *CmMYB1* negatively regulates flavonoids synthesis.

## Supporting Information

File S1 **Supporting information file containing the following files.** Table S1.Primer sequences used in this study. Table S2.Primer sequences used in qRT-PCR.
(DOC)
