# Supplementary material for: The Heterologous Expression of the Chrysanthemum R2R3-MYB Transcription Factor CmMYB1 Alters Lignin Composition and Represses Flavonoid Synthesis in Arabidopsis thaliana
Source: PLoS One. 2013 Jun 19;8(6):e65680. doi: 10.1371/journal.pone.0065680 (PMC3686752; doi:10.1371/journal.pone.0065680)
Supplement: File S1 — Supporting information file containing the following files. Table S1.Primer sequences used in this study. Table S2.Primer sequences used in qRT-PCR. (DOC) [file pone.0065680.s001.doc]

**Supplementary Table**

**Table S1**.**Primer sequences used in this study**

| Primer name | Sequences |
| --- | --- |
| F | 5’- AACAAAGGTGCATGGACCAAAG-3’ |
| R | 5’- ACACCTAAACTGCACGCAAAAC-3’ |
| oligo(dT) | 5’-GACTCGAGTCGACATCGATTTTTTTTTTTTTTTTT-3’ |
| dT-AP | 5’-GACTCGAGTCGACATCGA-3’ |
| GSP3’-1 | 5’- CAGACCATGTAGTCAAAAATGA-3’ |
| GSP3’-2 | 5’- AAGATGTCCAGATTTGAACTTG-3’ |
| AAP | 5’-GGCCACGCGTCGACTAGTACGGGIIGGGIIGGGIIG-3’ |
| AUAP | 5’-GGCCACGCGTCGACTAGTAC-3’ |
| GSP5’-1 | 5’- GTGGTGAGGTGACATTATGGT-3’ |
| GSP5’-2 | 5’- CTCGTTATCTGTTCTTCCTGG-3’ |
| GSP5’-3 | 5’- GGAGGCTGTGAAGTTTGATAA-3’ |
| Full-F | 5’- TGGGAAGTTCAAAGAAGAAAGTC-3’ |
| Full-R | 5’-CCAAATACATCTTACAAGCAATG-3’ |
| MYB1-RT-F | 5’- TAAGCTCAATATCACCTCGCCT-3’ |
| MYB1-RT-R | 5’- TTGTACACCTAAACTGCACGCA-3’ |
| CmGAPDH-F | 5’- CTGCTTCTTTCAACATCATTCC-3’ |
| CmGAPDH-R | 5’- CTGCTCATAGGTAGCCTTCTTC-3’ |
| MYB1-1301-F | 5’-GGATCCATGGGAAGGTCACCTTGTT-3’ |
| MYB1-1301-R | 5’-GAGCTCTTTCATCTCCAAGCTTCTA-3’ |

**Table S2.Primer sequences used in qRT-PCR.**

| Primer name | Gene AGI No. | Sequences |
| --- | --- | --- |
| AtCOMT1-F | [AT1G21100](http://www.arabidopsis.org/servlets/TairObject?id=136944&type=locus) | 5’- CGTCGCAGACAACTTTGATG-3’ |
| AtCOMT1-R | [AT1G21100](http://www.arabidopsis.org/servlets/TairObject?id=136944&type=locus) | 5’- TGATCTCCCACATGTCATCG-3’ |
| At4CL1-F | [AT1G51680](http://www.arabidopsis.org/servlets/TairObject?id=28261&type=locus) | 5’- CTCCGGTGTCTGGATCAACT-3’ |
| At4CL1-R | [AT1G51680](http://www.arabidopsis.org/servlets/TairObject?id=28261&type=locus) | 5’- GAAATCTGGTGCTGCTCCTC-3’ |
| AtC4H-F | [AT2G30490](http://www.arabidopsis.org/servlets/TairObject?id=35278&type=locus) | 5’- GCAAGCTGAATTGTCCACCT-3’ |
| AtC4H-R | [AT2G30490](http://www.arabidopsis.org/servlets/TairObject?id=35278&type=locus) | 5’- CACATCCTTGAAGCTGAGCA-3’ |
| AtCCoAOMT1-F | [AT4G34050](http://www.arabidopsis.org/servlets/TairObject?id=127920&type=locus) | 5’- CATCATCGACCAATGGAGAA-3’ |
| AtCCoAOMT1-R | [AT4G34050](http://www.arabidopsis.org/servlets/TairObject?id=127920&type=locus) | 5’- TCGATCAAACGCTTGTGGTA-3’ |
| AtCAD6-F | [AT4G37970](http://www.arabidopsis.org/servlets/TairObject?id=127437&type=locus) | 5’- CGAGTCTCTCAAACGCAGTG-3’ |
| AtCAD6-R | [AT4G37970](http://www.arabidopsis.org/servlets/TairObject?id=127437&type=locus) | 5’- GTTAGGTGGAGTCGGTCACA-3’ |
| AtUBQ10-F | [AT4G05320](http://www.arabidopsis.org/servlets/TairObject?id=126703&type=locus) | 5’- AGGACAAAGAGGGTATCCCA-3’ |
| AtUBQ10-R | [AT4G05320](http://www.arabidopsis.org/servlets/TairObject?id=126703&type=locus) | 5’-CAGACGCAAGACCAAGTGAA-3’ |
